# Fibrinogen *α*-chain-derived peptide is upregulated in hippocampus of rats exposed to acute morphine injection and spontaneous alternation testing

**DOI:** 10.1002/prp2.37

**Published:** 2014-04-07

**Authors:** Agatha E Maki, Kenneth A Morris, Kasia Catherman, Xian Chen, Nathan G Hatcher, Paul E Gold, Jonathan V Sweedler

**Affiliations:** 1 Beckman Institute, University of Illinois at Urbana-ChampaignUrbana, Illinois; 2 Neuroscience Program, University of Illinois at Urbana-ChampaignUrbana, Illinois; 3 Department of Chemistry, University of Illinois at Urbana-ChampaignUrbana, Illinois; 4 Department of Biology, Syracuse UniversitySyracuse, New York

**Keywords:** Fibrinogen, Fibrinogen *α*-chain peptides, fibrinopeptide A, hippocampus, in vivo microdialysis, mass spectrometry, morphine

## Abstract

Fibrinogen is a secreted glycoprotein that is synthesized in the liver, although recent in situ hybridization data support its expression in the brain. It is involved in blood clotting and is released in the brain upon injury. Here, we report changes in the extracellular levels of fibrinogen *α*-chain-derived peptides in the brain after injections of saline and morphine. More specifically, in order to assess hippocampus-related working memory, an approach pairing in vivo microdialysis with mass spectrometry was used to characterize extracellular peptide release from the hippocampus of rats in response to saline or morphine injection coupled with a spontaneous alternation task. Two fibrinopeptide A-related peptides derived from the fibrinogen *α*-chain – fibrinopeptide A (ADTGTTSEFIEAGGDIR) and a fibrinopeptide A-derived peptide (DTGTTSEFIEAGGDIR) – were shown to be consistently elevated in the hippocampal microdialysate. Fibrinopeptide A was significantly upregulated in rats exposed to morphine and spontaneous alternation testing compared with rats exposed to saline and spontaneous alternation testing (*P* < 0.001), morphine alone (*P* < 0.01), or saline alone (*P* < 0.01), respectively. The increase in fibrinopeptide A in rats subjected to morphine and a memory task suggests that a complex interaction between fibrinogen and morphine takes place in the hippocampus.

## Introduction

Fibrinogen is a plasma glycoprotein that is synthesized in the liver, secreted, and stored in *α*-granules of platelets and megakaryocytes (Handagama et al. 1989; Harrison et al. 1989). It is part of an extensive biochemical pathway involved in blood clotting and fibrinolysis. Fibrinogen is composed of two sets of three polypeptide chains A*α*, B*β*, and *γ*, which are joined by disulfide bridges at the N-terminal domain (Henschen et al. 1983; Zhang and Redman 1992; Huang et al. 1993). Each A*α*-chain contains an N-terminal fibrinopeptide A sequence, which is cleaved by thrombin and initiates fibrin assembly (Laudano and Doolittle 1980; Liu et al. 1985; Atkins et al. 1993). Fibrinopeptide A is a 17-amino acid peptide that has been shown to be an indicator of fibrin assembly/clot formation (Atkins et al. 1993). During blood coagulation, fibrinogen gets converted to fibrin by thrombin. Fibrin is then cross-linked with factor XIII to form a clot. During fibrinolysis, the opposite occurs; the enzyme plasmin (the zymogen form is plasminogen) helps dissolve the fibrin clots. Plasmin is activated by a variety of enzymes, such as tissue plasminogen activator (tPA), which binds to fibrin when plasminogen is activated (Hoylaerts et al. 1982).

Fibrinogen has predominantly been reported to be on the blood side of the blood–brain barrier and released into the brain upon injury (Adams et al. 2004; Ahn et al. 2010). However, in situ hybridization data from the Allen Mouse Brain Atlas show mRNA expression of fibrinogen *α-*, *β-*, and *γ*-chains in mouse brain, with the strongest expression in the hippocampus (©2012 Allen Institute for Brain Science. Allen Mouse Brain Atlas [Internet]. Available from: http://mouse.brain-map.org [Lein et al. 2007]). Furthermore, there are reports of interactions between fibrinogen and morphine. Buczko and Wiśniewski (1972) have shown that fibrinogen degradation products can potentiate morphine’s analgesic effects. In addition, morphine therapy after surgery can lead to increased blood clotting and consequently, arterial thrombosis (Rosenfeld et al. 1993).

While direct studies of a preselected peptide such as fibrinopeptide A use selective approaches like immunohistochemistry, mass spectrometry (MS)-based studies allow detection of unspecified brain peptides (Che et al. 2001; Dowell et al. 2006; Li and Sweedler 2008; Bernay et al. 2009). Prior investigations using MS have identified hundreds of prohormone-derived and other protein-derived peptides in the brain, and a subset of these studies have detected fibrinogen-related peptides (Dowell et al. 2006; Hatcher et al. 2008; Zhang et al. 2008; Lee et al. 2010). Besides characterizing the peptides in a tissue, sampling peptide release from the brain is important for functional studies, and can involve sampling release from cultured cells and brain slices (Hatcher et al. 2008; Croushore and Sweedler 2013). Perhaps the best known approach for sampling brain neurochemistry is microdialysis (Ao and Stenken 2006; Schultz and Kennedy 2008; Bernay et al. 2009).

Previous work has shown that spontaneous alternation tasks can be used to test for memory deficits. These are hippocampus-sensitive tasks that measure spatial working memory. Memory scores on these and other memory tests are impaired by opioid receptor agonists (Costa et al. 1983; Bostock et al. 1988; Ragozzino et al. 1992; Gorman et al. 1994; Ragozzino and Gold 1995; Wan et al. 1995; McNay and Gold 1998; McNay et al. 2006); opioid agonists also impair release of at least one neurotransmitter, acetylcholine, in a manner related to the memory impairments (Costa et al. 1983; Bostock et al. 1988; Ragozzino et al. 1992; Gorman et al. 1994; Ragozzino and Gold 1995; Wan et al. 1995; McNay and Gold 1998; McNay et al. 2006).

Here, we used in vivo microdialysis paired with MS to examine the effects of morphine on peptide release in the hippocampus. We detected two peptides derived from the fibrinogen *α*-chain that were increased in the microdialysate. One of the peptides was fibrinopeptide A (*m/z* 1738.80); the other was a fibrinopeptide A-derived peptide (*m/z* 1667.76) that results from the cleavage between amino acids A and D in the N-terminus. We used Fourier transform ion cyclotron resonance mass spectrometry (FT-ICR MS, or FTMS) to sequence these peptides. In particular, fibrinopeptide A was significantly upregulated in rats that had been given a morphine injection and placed on a four-arm radial maze to perform a spontaneous alternation task compared with rats given a saline injection and exposed to a spontaneous alternation task (*P* < 0.001), saline injection alone (*P* < 0.01), or morphine injection alone (*P* < 0.01), respectively. These results suggest an interaction between fibrinogen and morphine in the brain.

## Materials and Methods

### Animals

Adult male Sprague–Dawley rats were housed in the animal facility at the Psychology Building (University of Illinois at Urbana-Champaign). The animals were singly housed in translucent cages maintained under controlled environmental conditions, with standard 12-h alternating periods of light and dark. Rodent chow and water were provided ad libitum. Rats were handled daily for 5 min/day starting at least 7 days before the behavioral experiments. Pain and discomfort were minimized throughout the study. Animal care, euthanasia, and other experimental procedures were in accordance with the Principles of Laboratory Animal Care (NIH Publication no. 85-23) and protocols approved by the University of Illinois at Urbana-Champaign Institutional Animal Care and Use Committee; in addition, the ARRIVE guidelines were followed. A total of 22 rats were used to optimize procedures, after which, 15 rats were used for the in vivo microdialysis experiments as described below.

### In vivo microdialysis

Rats were anesthetized with isoflurane and placed in a stereotaxic apparatus. CMA/12 guide cannulae (CMA Microdialysis AB, Kista, Sweden) were bilaterally implanted above the central portion of the ventral hippocampus (coordinates: –5.5 mm from the bregma; ±4.8 mm lateral; –4.2 mm deep from the skull). Skull screws were inserted and the entire assembly was anchored in place with dental cement. Rats were monitored and allowed to recover for approximately 1 week. On the day of in vivo microdialysis collections, 3-mm CMA/12 microdialysis probes (CMA Microdialysis AB) with 20 kDA cut-off membranes were inserted into the ventral hippocampi. Brains were perfused continuously with artificial cerebrospinal fluid (128 mmol/L NaCl, 2.5 mmol/L KCl, 1.3 mmol/L CaCl_2_, 2.1 mmol/L MgCl_2_, 0.9 mmol/L NaH_2_PO_4_, 2.0 mmol/L Na_2_HPO_4_, 1.0 mmol/L dextrose, pH 7.4) at a rate of 2 *μ*L/min. A constant perfusion rate throughout the in vivo microdialysis experiments was achieved by an automated CMA/100 microinjection pump (CMA Microdialysis AB).

In order to optimize the microdialysis sampling and MS measurements, 22 rats were used to determine the optimal intervals at which to collect release, ensure consistent results, and validate our ability to characterize peptides. Analysis of releasates from these animals collected every 10 min for 120 min provided time course information and significant peptide peak intensities. Additionally, left and right microdialysis probes from the same rat brain samples were tested for reproducibility of results, and the peptide profiles were found to be similar. These preliminary experiments used *n* = 4, morphine; *n* = 5, saline; *n* = 8, morphine-testing; *n* = 5, saline-testing animals (see below for details on the injections); because the procedures were being optimized with these experiments, including a variety of collection intervals, the data are not shown nor were they used during subsequent measurements.

For the final experiments, rats received either a single subcutaneous morphine injection (5 mg/kg) or saline injection (*n* = 4, morphine; *n* = 3, saline). Samples were collected every 10 min before, during, and after injection for a total of 120 min. In a subsequent experiment, rats received either an acute subcutaneous morphine injection (5 mg/kg) or saline injection and were then placed in a four-arm radial maze for 20 min for spontaneous alternation testing (*n* = 3, morphine-testing; *n* = 4, saline-testing). Samples were collected every 10 min before, during, and after injection and testing, for a total of 120 min. Samples collected during the first hour of microdialysis were discarded to allow for baseline stabilization (Westerink and Timmerman 1999). One additional rat (*n* = 1) was used for an extended collection of in vivo microdialysate for peptide sequencing analysis as described below. The peptides were concentrated by solid phase extraction via pre-equilibrated C18 ZipTip pipette tips (Millipore, Billerica, MA) in line with the perfusate output. The efficacy of ZipTip pipette tips to capture peptides has previously been shown (Baczek 2004; Wa et al. 2006); we have optimized a variety of solid phase extraction protocols to ensure high efficiency collection of neuropeptides (Hatcher et al. 2008; Iannacone et al. 2009; Annangudi et al. 2010). Each ZipTip was eluted in 0.3 *μ*L increments of 70% acetonitrile (ACN) onto five spots on a Prespotted AnchorChip (PAC) target (Bruker Daltonics, Billerica, MA) containing *α*-cyano-4-hydroxy-cinamic acid (HCCA) as the matrix-assisted laser desorption/ionization (MALDI) matrix. To ensure consistency across rats and experimental sampling, the in vivo microdialysis experiments were started between 8 and 9 AM to reduce changes that might be based on circadian rhythms.

Probe placements in the ventral hippocampi were previously verified with histology using another group of rats, with no errors found (Morris et al. 2010). Briefly, rats were deeply anesthetized with an overdose intraperitoneal injection of sodium pentobarbital (Sigma-Aldrich, St. Louis, MO) and then perfused intracardially with 80 mL of 0.1 M phosphate-buffered saline followed by 80 mL of 4% paraformaldehyde in 0.1 M phosphate buffer. Rats were then decapitated and the brains removed and placed into 4% paraformaldehyde in 0.1 M phosphate buffer for ∼72 h. The brains were transferred to a solution of 20% glycerol in 0.1 M phosphate-buffered saline and stored for ∼48 h. Frozen sections (40 *μ*mol/L) containing the microdialysis probe tracts were collected at –30 ^o^ C with a Leica 1800 cryostat (Leica Microsystems, Wetzlar, Germany). Sections were mounted onto gelatin-coated slides and stained with cresyl violet to visualize probe placements using a dissection microscope.

### Spontaneous alternation testing

**Figure 1:**
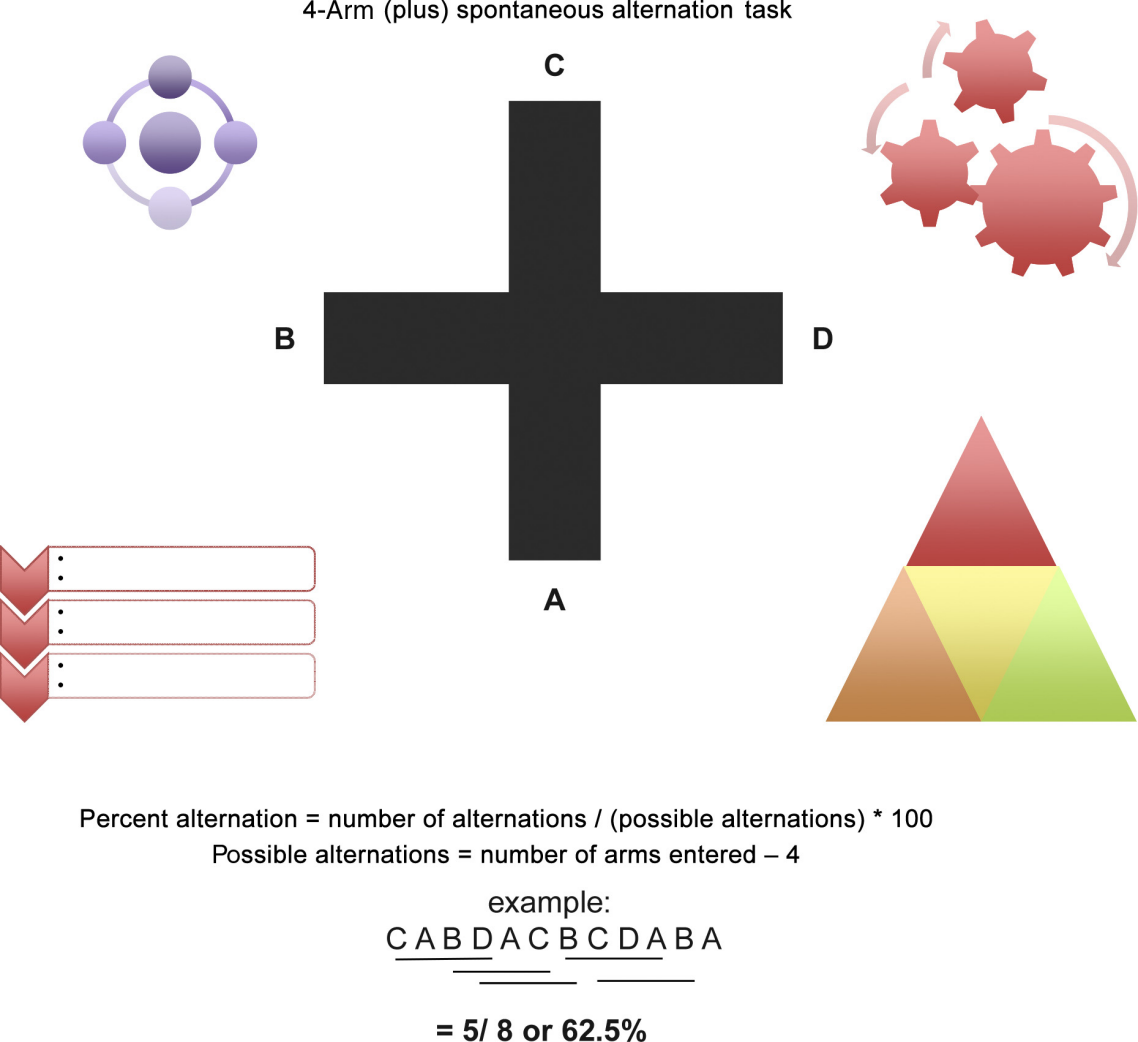
Schematic of the spontaneous alternation task. Graphics outside of maze represent two- and three-dimensional extramaze cues (e.g., posters, shelves, file cabinet, door) in the testing room. Rats are placed in a start arm and permitted to explore for 20 min. Memory is assessed as a percent of the alternation scores as shown below the maze. In the experiments presented here, an alternation was defined as entry into four different arms during five consecutive entries (underlined in example). Each set of five consecutive arm entries used for calculation of the alternation score was continuously overlapping with the next, that is, four different arms in entries 1–5, 2–6, 3–7, etc.

Spontaneous alternation performance was assessed in a four-arm plus-shaped black plexiglass maze with an open ceiling (Fig. 1). The dimensions of each arm were 45 cm L × 13 cm W × 7 cm H. The dimensions of the central square-shaped area were 13 cm L × 13 cm W × 7 cm H. Rats were placed in a random start arm and allowed to freely traverse the maze for 20 min. The numbers and sequences of entries were recorded. All behavioral testing was performed during the morning hours to reduce changes that might be based on circadian rhythms and thus, reduce variability across experiments and rats.

Spontaneous alternation tasks are standard tests of spatial working memory (reviewed in Lalonde 2002), used often in pharmacological studies of memory including in combination with microdialysis methods (Ragozzino et al. 1996; McNay et al. 2000, 2001; Hughes 2004; Chang and Gold 2008; De Bundel et al. 2009). For the experiments described here, the task involved placing the rat in a start arm and permitting it to roam through the maze for 20 min. Rats, like other mammals, tend to move through the maze in a nonrandom manner. Specifically, the rats alternate arm choices so that they balance the most and least recently visited arms. For example, in the four-arm maze used here, if a rat has visited arms A, D, B, it will be likely to visit C next, which would complete an alternation pattern that includes all four arms. Based on extensive prior experience with this task, we define an alternation as entries into four different arms across a set of five consecutive arm entries. Thus, ABDCB, but not ABDAB, would be counted as an alternation. Using this procedure, the total number of possible alternations is equal to the number of arm entries minus 4. The percent alternation score is equal to actual alternations/possible alternations ×100. Using this calculation, chance performance is 44%. In control rats, typical alternation scores are about 60%, revealing the tendency to enter the least recently visited arm. This tendency is the basis for the memory load in this task as the rat must remember its recent entry pattern in order to perform at levels above chance, in part using extramaze cues as the basis for spatial navigation; alternation scores decline toward chance values if rats are tested in a room with poor cue availability. For this reason, the training room contained numerous extramaze visual cues in the form of objects and wall decorations.

### Analysis of microdialysate by MS

Mass spectrometric analysis of the samples was performed using a MALDI time-of-flight/time-of-flight mass spectrometer (ultrafleXtreme; Bruker Daltonics) using methods based on the protocols we have optimized for neuropeptide characterization (Hummon et al. 2006; Rubakhin and Sweedler 2008; Hou et al. 2012). Here, the samples were eluted onto PAC targets prespotted with calibration spots containing standard peptides (bradykinin 1-7, angiotensin II, angiotensin I, substance P, bombesin, ACTH clip 1-17, ACTH clip 18-39, somatostatin 28) in the mass range of 750–3100 Da to externally calibrate the instrument. Mass spectra were obtained in reflectron mode within 100 ppm mass accuracy in the mass range of *m/z* 900–4000. For each of the microdialysis releasate fractions (five fractions/spots per ZipTip), data were collected using the AutoExecute™ function of the ultrafleXtreme mass spectrometer with automated acquisition of spectra by panning across the spot with a total of 1000 laser shots (sum of 10 × 100 laser shots) and combing the spectra. Using the flexAnalysis 3.3 software package (Bruker Daltonics), mass spectra were smoothed and baseline corrected, and peaks with a signal-to-noise ratio greater than 3:1 were analyzed for intensity.

### Peptide extraction and sequencing analysis by FTMS

Microdialysate collected over 3 h on two separate days from a single morphine-injected rat (*n* = 1) was subjected to sequencing analysis. Samples were collected at 1-h intervals using pre-equilibrated C18 ZipTip pipette tips and eluted with 0.5 *μ*L of 70% ACN into low-bind tubes (Sigma-Aldrich). Tube contents from each 1 h collection were then combined, dried with a Savant SpeedVac Vacuum Concentrator (Thermo Scientific, Waltham, MA), and reconstituted in 100 *μ*L of deionized water. The reconstituted samples were desalted using a C18 spin column (Pierce, Rockford, IL). The eluted samples from the spin column were dried using the SpeedVac and then reconstituted in 20 *μ*L of 95/5 H_2_O/ACN and analyzed with an 11 Tesla linear trap quadrupole-FT mass spectrometer (LTQ-FT Ultra; ThermoFisher Scientific, San Jose, CA) equipped with a nanoscale high performance liquid chromatography pump (NanoLC-1D; Eksigent Technologies, Dublin, CA) (Lee et al. 2010, 2013). The flow rate was 300 nL/min and the gradient used was 0–35 min, 1–20% B; 35–55 min, 20–55% B; 55–60 min, 55–95% B; 60–63 min, 95–95% B; 63–68 min, 95–5% B; 68–80 min, 5–5% B. The LTQ-FTMS analysis included a full scan event and data-dependent collision-induced dissociation tandem MS (MS/MS) scans of the three most abundant peaks from the full scan. The resulting liquid chromatography-FTMS/MS spectra were searched against an intact rat database in a “neuropeptide” search mode using ProSight PC 2.0 (ThermoFisher Scientific), with tolerances of ±1.1 Da and ±10 ppm for intact mass and fragment, respectively. The FTMS methodology used here followed closely what was previously reported for neuropeptide characterization (Lee et al. 2010, 2013).

### Statistics

Statistical analyses were conducted using StatView statistical software (SAS Institute Inc., Cary, NC). Student’s *t*-tests were performed to determine the statistical significance of the spontaneous alternation performance and arms visited. Repeated measures of analysis of variance (ANOVA) were conducted for group effects of peak intensity across collection time points. Post hoc analyses were conducted using the Fisher’s protected least significant difference (PLSD) test. For these measurements, *P* < 0.05 was considered statistically significant.

## Results

**Figure 2:**
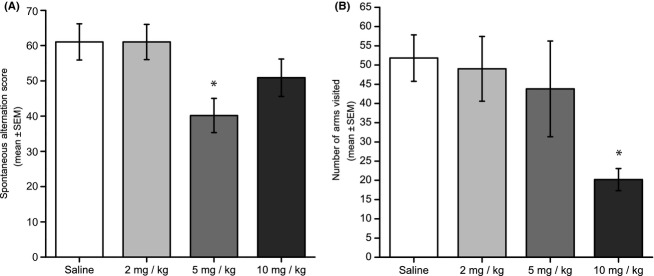
Dose–response curve for morphine effects on spontaneous alternation and on number of arms visited. Rats were subjected to the spontaneous alternation parameters as described in Figure 1. (A) Spontaneous alternation scores from rats given an acute intraperitoneal injection of saline (*n* = 5), 2 mg/kg morphine (*n* = 5), 5 mg/kg morphine (*n* = 5), and 10 mg/kg morphine (*n* = 5). The 5 mg/kg morphine dose impaired the spontaneous alternation score (*P < 0.05). (B) Number of arms visited from rats given an acute injection of saline (*n* = 5), 2 mg/kg morphine (*n* = 5), 5 mg/kg morphine (*n* = 5), and 10 mg/kg morphine (*n* = 5). The 5 mg/kg morphine dose did not affect arm entries or motor activity during spontaneous alternation. The 10 mg/kg morphine dose significantly reduced arm entries (*P < 0.05). Error bars represent SEM.

An initial dose–response experiment revealed alternation scores lower than those of controls in rats treated with 5 mg/kg, but not with 2 or 10 mg/kg morphine (Fig. 2A, B; *P* < 0.05). The dose-range was based on past experiments of morphine impairment of memory (Izquierdo 1979; Izquierdo et al. 1980; Stone et al. 1991; Braida et al. 1994; Khajehpour et al. 2008; Farahmandfar et al. 2010). Studies of doses near these amounts indicated that systemically administered morphine reaches peak levels in blood and brain in about 10 min, with a half-life of about 30–45 min in blood and brain (Bouw et al. 2000). These results are consistent with previous work showing that morphine has a deleterious effect on learning and memory in several tasks, including tests of spontaneous alternation (Bostock et al. 1988; Ragozzino et al. 1992; Ragozzino and Gold 1995; Wan et al. 1995; McNay and Gold 1998; McNay et al. 2006). The maximally effective dose varies with task, among other variables, across studies and the 5 mg/kg dose identified here is within a typical range of effective doses in the experiments cited above. In some instances, dose–response curves for impairments of memory are a nonmonotonic function as seen here (Farahmandfar et al. 2010). The memory-impairing dose did not significantly affect the total number of arm entries, although there was a decrease in arm entries at the high (10 mg/kg) dose.

**Figure 3:**
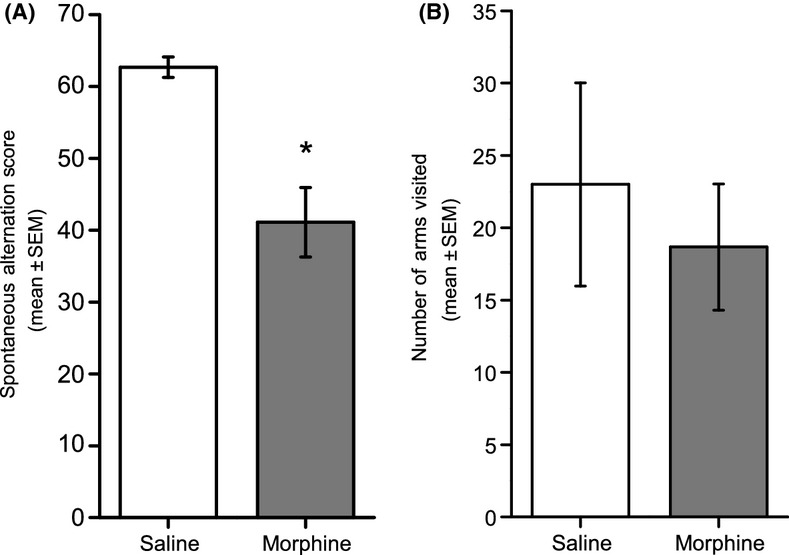
Effects of acute injection of 5 mg/kg morphine on spontaneous alternation and number of arms visited in rats undergoing in vivo microdialysis. Rats were subjected to the same spontaneous alternation parameters as described in Figure 1, in addition to having in vivo microdialysis probes inserted into the ventral hippocampus. (A) Spontaneous alternation scores from rats given an acute injection of saline (*n* = 4) or 5 mg/kg morphine (*n* = 3). Scores from rats treated with morphine are significantly reduced (*P < 0.05). (B) Number of arms visited from rats given an acute injection of saline (*n* = 4) or 5 mg/kg morphine (*n* = 3). Morphine did not affect arm entries. Error bars represent SEM.

In vivo microdialysis with MS was used to characterize extracellular peptide level changes in the hippocampus of awake and freely moving rats in response to acute subcutaneous morphine injection (5 mg/kg), saline injection, morphine injection (5 mg/kg) paired with spontaneous alternation, and saline injection paired with spontaneous alternation, respectively. In this microdialysis experiment, morphine (5 mg/kg; *n = 3*) again resulted in alternation scores lower than those of saline-treated controls (*n = 4*) and the morphine-treated rats again exhibited total arm entries comparable to those of controls (Fig. 3A, B; *P* < 0.05).

**Figure 4:**
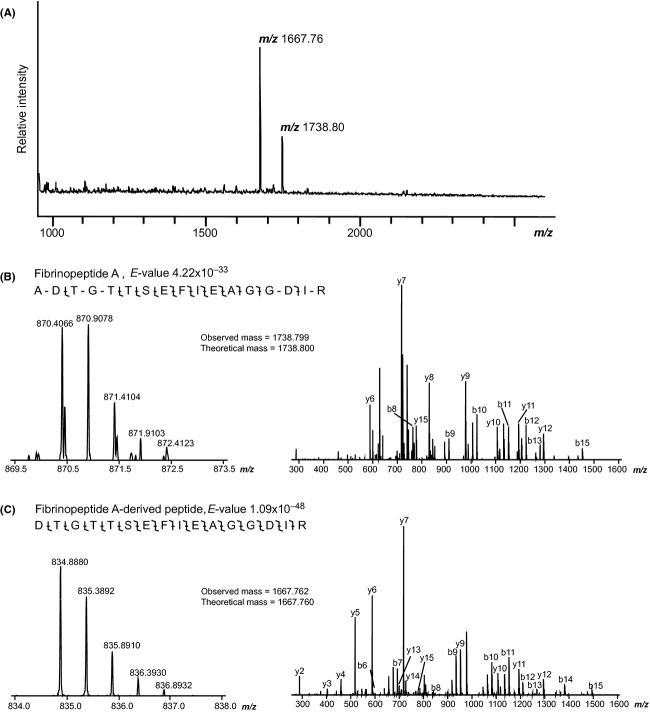
Sequencing of fibrinogen *α*-chain-derived peptides. Microdialysate was collected for 3 h from a rat injected with morphine and tested on a spontaneous alternation task. The LTQ-FT Ultra mass spectrometer was used to sequence the particular peaks observed with high intensity in all animal groups and sample collections. (A) MALDI-MS spectrum showing peaks *m/z* 1738.80 and *m/z* 1667.76, consistently detected throughout the microdialysis collections. (B) FTMS and FTMS/MS spectra of fibrinopeptide A (ADTGTTSEFIEAGGDIR), *m/z* 1738.799, which was identified with 7 b-ions and 8 y-ions with high confidence (*E* value of 4.22 × 10^−33^). Fibrinopeptide A was found to be derived from the fibrinogen *α*-chain. (C) FTMS and FTMS/MS spectra of a fibrinopeptide A-derived peptide (DTGTTSEFIEAGGDIR), *m/z* 1667.762, identified with 14 b-ions and 12 y-ions with high confidence (*E* value of 1.98 × 10^−48^). The fibrinopeptide A-derived peptide was found to be derived from the fibrinogen *α*-chain.

**Figure 5:**
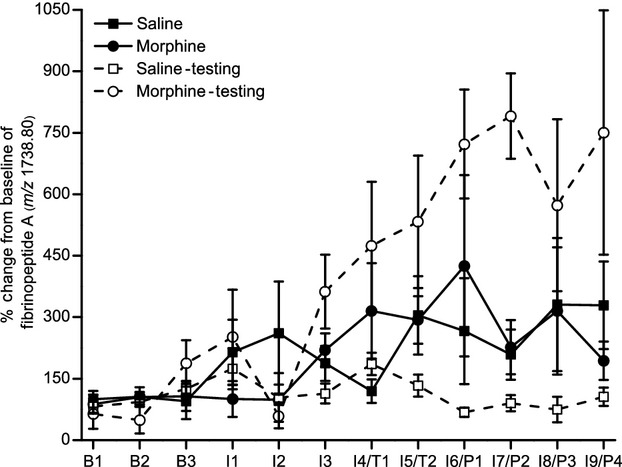
Percent change from baseline of fibrinopeptide A (*m/z* 1738.80) intensity. Rats received either an acute subcutaneous morphine injection (5 mg/kg) or saline injection (*n* = 4, morphine; *n* = 3, saline). In a subsequent experiment, rats received either an acute subcutaneous morphine injection (5 mg/kg) or saline injection, and were additionally placed in a four-arm radial maze for 20 min for spontaneous alternation testing (*n* = 3, morphine-testing; *n* = 4, saline-testing). In vivo microdialysate was collected every 10 min before, during, and after injection/testing, and analyzed by MS. Time collections are labeled as baseline (B), injection (I), testing (T), and posttesting (P), and each label corresponds to a consecutive 10 min collection. Time collections I4/T1, I5/T2, I6/P1, I7/P2, I8/P3, and I9/P4 have been grouped together on the graph as they correspond to the same time points in different experimental groups. The percent change from baseline was calculated by dividing the intensity of fibrinopeptide A at each particular time point by the average of the intensity of the first three baseline time points (B1, B2, B3). Repeated measures ANOVA showed significant group differences (comparing saline, morphine, saline-testing, morphine-testing groups) in percent change from baseline of fibrinopeptide A intensity (*P* < 0.01). There were post hoc effects showing increased intensity in the morphine-testing group (*n* = 3) compared to the saline group (*n* = 3; *P* < 0.01), saline-testing group (*n* = 4; *P* < 0.001), and morphine group (*n* = 4; *P* < 0.01). TreatmentGroup: *P* < 0.01; Microdialysis: *P* < 0.001; Microdialysis * TreatmentGroup: *P* < 0.05. Error bars represent SEM.

**Figure 6:**
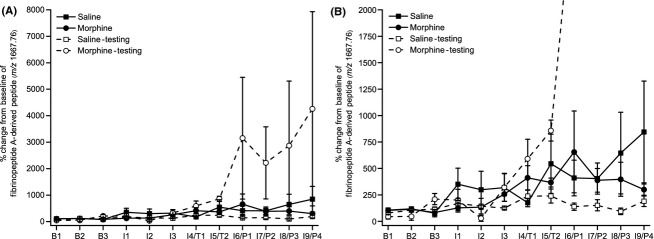
Percent change from baseline of fibrinopeptide A-derived peptide (*m/z* 1667.76) intensity. Rats were subjected to the same experimental design as in Figure 4. The percent change from baseline was calculated by dividing the intensity of fibrinopeptide A-derived peptide at each particular time point by the average of the intensity of the first three baseline time points (B1, B2, B3). Repeated measures ANOVA did not show any group differences (comparing saline, morphine, saline-testing, morphine-testing groups) in percent change from baseline of fibrinopeptide A-derived peptide intensity (*P* = 0.146). There was a post hoc effect showing increased intensity in the morphine-testing group (*n* = 3) as compared to the saline-testing group (*n* = 4; *P* < 0.05). (A) A zoomed-out version of the graph and (B) a zoomed-in version of the graph. TreatmentGroup: *P* = 0.146; Microdialysis: *P* = 0.0579; Microdialysis × TreatmentGroup: *P* = 0.1867. Error bars represent SEM.

Two peaks (*m/z* 1667.76 and 1738.80) were consistently detected throughout the microdialysis collections in the four animal groups: (1) saline (*n* = 3), (2) morphine (*n* = 4), (3) saline-testing (*n* = 4), (4) morphine-testing (*n* = 3) (Fig. 4A). The peaks were sequenced using a longer (3 h) microdialysate collection to collect a greater amount of peptide in order to have enough peptide for sequencing. We confirmed that these masses correspond to two peptides derived from the fibrinogen *α*-chain: (1) fibrinopeptide A with *m/z* 1738.80 (Fig. 4B) and (2) a fibrinopeptide A-derived peptide with *m/z* 1667.76, missing the N-terminal residue as a result of cleavage between amino acids A and D at the N-terminus (Fig. 4C). Others have also identified these masses as corresponding to fibrinogen *α*-chain peptides (Dowell et al. 2006). The peak intensity for each of the peaks was obtained for each collection time point. Repeated measures ANOVA showed significant group differences (comparing saline, morphine, saline-testing, morphine-testing groups) in percent change from the baseline of fibrinopeptide A intensity, *F*(3, 10) = 10.563, *P* < 0.01 (Fig. 5). Fisher’s PLSD also revealed post hoc effects showing increased intensity in the morphine-testing group (*n* = 3) compared with the saline group (*n* = 3; *P* < 0.01), saline-testing group (*n* = 4; *P* < 0.001), and morphine group (*n* = 4; *P* < 0.01), respectively. There were significant differences across microdialysis collection time points (microdialysis; *P* < 0.001) and significant interaction between treatment groups and microdialysis collection points (microdialysis × treatment group; *P* < 0.05). Repeated measures ANOVA did not show any group differences (comparing saline, morphine, saline-testing, morphine-testing groups) in percent change from the baseline of fibrinopeptide A-derived peptide intensity, *F*(3, 10) = 2.247, *P* = 0.146 (Fig. 6A, B [zoomed-in]). However, Fisher’s PLSD revealed a post hoc effect showing increased intensity in the morphine-testing group (*n* = 3) compared with the saline-testing group (*n* = 4; *P* < 0.05). A control peak at *m/z* 987.53, which was de novo sequenced (Fig. S1A, B), was also analyzed in greater detail between all four groups. Data from one rat in the morphine-testing group were excluded from the control peak analysis because they did not contain the particular peak of interest. Repeated measures ANOVA did not reveal any group differences (comparing saline, morphine, saline-testing, morphine-testing groups) in percent change from the baseline of peak *m/z* 987.53 intensity (Fig. S2; *P* = 0.297). These results show that the changes observed for the fibrinogen *α*-derived peptides do not represent an overall increase in peptides and the changes are peptide specific. While other peptide peaks were detected, these peaks were not observed consistently across rats or conditions.

## Discussion

Here, we have observed the effects of morphine on changes in extracellular peptide levels from the hippocampus utilizing in vivo microdialysis paired with MS. While many peaks were observed inconsistently, we found several that were observed consistently. A fibrinogen *α*-chain-derived peptide, fibrinopeptide A, was upregulated in response to morphine and spontaneous alternation. Although this appears to be the first report of fibrinogen level changes in rat brain, human studies have shown that opium users have higher fibrinogen blood concentrations (Galante et al. 1994; Asgary et al. 2008). In situ hybridization data from the Allen Mouse Brain Atlas show mRNA expression of fibrinogen chains in the mouse brain. Although these recent data suggest that fibrinogen is synthesized in the brain, further analysis is needed to confirm the expression, synthesis, and release of fibrinogen from neurons. Conversely, there are reports stating that fibrinogen is neither synthesized nor present in the brain as the blood–brain barrier excludes it (Adams et al. 2004; Ahn et al. 2010). Furthermore, fibrinogen has been found in damaged vasculature/brain tissue and, in particular, accumulates in Alzheimer’s disease (Paul et al. 2007). Amyloid-beta interacts with fibrinogen and causes the formation of abnormally strong fibrin clots, a possible cause of the high number of blocked blood vessels that occurs in Alzheimer’s patients (Ahn et al. 2010; Cortes-Canteli et al. 2010). In addition, fibrinogen appears to be a mediator of inflammation at the blood–brain barrier; thus, the injury caused by insertion of the microdialysis probes combined with the physical activity in our study might be one factor causing its levels to increase. However, after administration of a memory-impairing dose (5 mg/kg) of morphine as used in the microdialysis experiments, the locomotor activity of rats, assessed as total arm entries on the spontaneous alternation task, did not differ significantly from that of saline-injected rats, even though peptide release increased. Conversely, the observed increases in the levels of fibrinogen might indicate that it influences the effects of morphine. Buczko and Wiśniewski (1972) have shown that trypsin-digested fibrinogen degradation products can potentiate the actions of morphine. They also determined that degradation products smaller than approximately *m/z* 1500 actually abolished morphine’s analgesic activity (Buczko and Wiśniewski 1972).

It is also possible that morphine is directly involved in the increased levels of the fibrinogen *α*-chain-derived peptides we observed. Morphine has been reported to alter the release of neurotransmitters in the hippocampus, but there appear to be no reports of its effects on peptide release in the hippocampus. Previous studies have shown that intraseptal injections of opioid agonist can decrease the release of acetylcholine from the hippocampus (Costa et al. 1983; Gorman et al. 1994; Ragozzino and Gold 1995). In addition, in the medial septum, activation of opioid receptors seems to inhibit GABAergic interneurons that influence both cholinergic and GABAergic neurons projecting to the hippocampus (Alreja et al. 2000).

Furthermore, although we show that one fibrinogen *α*-chain peptide is upregulated with an acute morphine injection and spontaneous alternation, it is reasonable to speculate that other proteins in the blood clotting pathway are possibly affected. As one example, tPA, involved in fibrinolysis, is synthesized and released by neurons and has been shown to aid in sympathetic neuronal function and transmitter release (Wu et al. 2000; Jacovina et al. 2001; Schaefer et al. 2006). tPA is mostly synthesized by endothelial cells and is found in blood (Levin 1983). By activating plasmin, tPA plays an indirect role in the cleavage of pro–brain-derived neurotrophic factor (proBDNF) into mature BDNF (mBDNF) (Pang et al. 2004). tPA itself is unable to cleave proBNDF; however, tPA together with plasminogen has been shown to be as effective as plasmin in generating mBDNF, suggesting that tPA affects proBDNF cleavage indirectly by activating plasmin (Pang et al. 2004). Perhaps tPA is upregulated in the hippocampus as well, which could in turn upregulate BDNF.

The possible relationship with BDNF is of particular interest because of its role in many brain processes, including neuronal survival and differentiation (Alderson et al. 1990; Nonomura et al. 1995; Abiru et al. 1996; Nonner et al. 1996; Ha et al. 1999), synaptic plasticity (Poo 2001), and long-term potentiation (Patterson et al. 1992; Kang et al. 1997). It is housed in large dense core vesicles (Fawcett et al. 1997; Michael et al. 1997; Haubensak et al. 1998; Möller et al. 1998; Luo et al. 2001) and is released both via the constitutive and regulated secretory pathways (reviewed in Lessman et al. 2003). Interestingly, BDNF has also been shown to upregulate peptide mRNA synthesis and release in the cortex and hippocampus (Nawa et al. 1994; Marty et al. 1997). Future work will determine whether morphine, in combination with a spontaneous alternation task, upregulates tPA activity and leads to more synaptic plasticity changes associated with BDNF.

In conclusion, morphine combined with a spontaneous alternation task significantly increased the extracellular levels of fibrinopeptide A in the hippocampus. The “local” increase in specific fibrinogen-related peptides in the brain is intriguing, and the interplay between fibrinogen and morphine remains to be elucidated.

## Abbreviations

ACN: acetonitrile
FTMS: Fourier transform mass spectrometry
HCCA: *α*-cyano-4-hydroxy-cinamic acid
MALDI: matrix-assisted laser desorption/ionization
mBDNF: mature brain-derived neurotrophic factor
MS/MS: tandem mass spectrometry
MS: mass spectrometry
PAC: Prespotted AnchorChip
PLSD: protected least significant difference
proBDNF: pro–brain-derived neurotrophic factor
tPA: tissue plasminogen activator

## Supporting Information

**Figure S1.** FTMS analysis of unknown *m/z* 987.53, a mass peak observed in 13 of the total 14 rats analyzed. Microdialysate was collected for 3 h from a rat injected with morphine and tested on a spontaneous alternation task. FTMS was used to sequence this particular peak observed with high intensity throughout animal groups and sample collections. (A) FTMS spectrum showing an unknown peptide. (B) FTMS/MS spectrum showing that this unknown peptide was unable to be identified as a peptide derived from any known prohormone precursors. **Figure S2.** Percent change from baseline of *m/z* 987.53 intensity. Rats were subjected to the same experimental design as in Figures 4 and 5. The percent change from baseline was calculated by dividing the intensity of the unknown at each particular time point by the average of the intensity of the first three baseline time points (B1, B2, B3). Repeated measures ANOVA showed no significant group differences (comparing saline, morphine, saline-testing, morphine-testing groups) in percent change from baseline of unknown intensity (*n* = 3, saline; *n* = 3, morphine; *n* = 4, saline-testing; *n* = 3, morphine-testing; *P* = 0.297). Error bars represent SEM.
