# Supplementary material for: Fibrinogen α-chain-derived peptide is upregulated in hippocampus of rats exposed to acute morphine injection and spontaneous alternation testing
Source: Pharmacol Res Perspect. 2014 Apr 7;2(3):e00037. doi: 10.1002/prp2.37 (PMC4024393; doi:10.1002/prp2.37)
Supplement: Supplementary file 1 [file prp20002-e00037-SD1.pdf]

A Fibrinogen-α Chain-derived Peptide is Upregulated in Hippocampus of Rats Exposed to Acute Morphine Injection and Spontaneous Alternation Testing

Agatha E. Maki, Kenneth A. Morris, Kasia Catherman, Xian Chen, Nathan G. Hatcher, Paul E. Gold, Jonathan V. Sweedler

Supplemental Figures

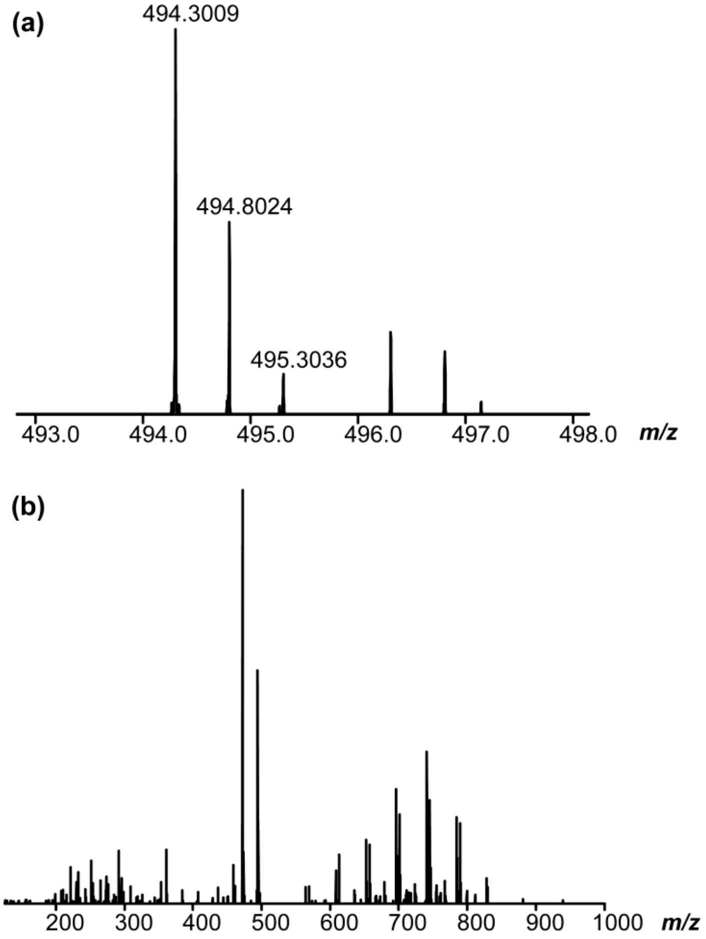

Fig. S1. FTMS analysis of unknown *m/z* 987.53, a mass peak observed in 13 out of the total 14 rats analyzed. Microdialysate was collected for 3 h from a rat injected with morphine and tested on a spontaneous alternation task. FTMS was used to sequence this particular peak observed with high intensity throughout animal groups and sample collections. (**a**) FTMS spectrum showing an unknown peptide. (**b**) FTMS/MS spectrum showing that this unknown peptide was unable to be identified as a peptide derived from any known prohormone precursors.

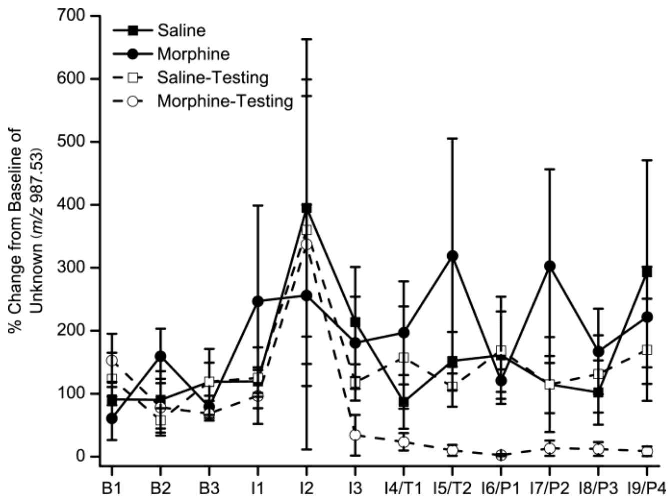

Fig. S2. Percent change from baseline of *m/z* 987.53 intensity. Rats were subjected to the same experimental design as in Figs. 4 and 5. The percent change from baseline was calculated by dividing the intensity of the unknown at each particular time point by the average of the intensity of the first three baseline time points (B1, B2, B3). Repeated measures ANOVA showed no significant group differences (comparing saline, morphine, saline-testing, morphine-testing groups) in percent change from baseline of unknown intensity (n = 3, saline; n = 3, morphine; n = 4, saline-testing; n = 3, morphine-testing; p = 0.297). Error bars represent SEM.
